# The Ghrelin/Growth Hormone Secretagogue Receptor System Is Involved in the Rapid and Sustained Antidepressant-Like Effect of Paeoniflorin

**DOI:** 10.3389/fnins.2021.631424

**Published:** 2021-02-16

**Authors:** Yuan Zhang, Min-Zhen Zhu, Xi-He Qin, Yuan-Ning Zeng, Xin-Hong Zhu

**Affiliations:** ^1^Institute of Mental Health, School of Basic Medical Science, Southern Medical University, Guangzhou, China; ^2^Key Laboratory of Mental Health of the Ministry of Education & Guangdong Province Key Laboratory of Psychiatric Disorders, Guangzhou, China; ^3^Guangdong-Hong Kong-Macao Greater Bay Area Center for Brain Science and Brain-Inspired Intelligence, Guangzhou, China; ^4^Eusyn Medical Technology Company, Guangzhou, China; ^5^School of Psychology, Shenzhen University, Shenzhen, China

**Keywords:** major depressive disorder, paeoniflorin, growth hormone secretagogue receptor 1α, intestine, antidepressant

## Abstract

Major depressive disorder (MDD) is a debilitating mental illness affecting people worldwide. Although significant progress has been made in the development of therapeutic agents to treat this condition, fewer than half of all patients respond to currently available antidepressants, highlighting the urgent need for the development of new classes of antidepressant drugs. Here, we found that paeoniflorin (PF) produced rapid and sustained antidepressant-like effects in multiple mouse models of depression, including the forced swimming test and exposure to chronic mild stress (CMS). Moreover, PF decreased the bodyweight of mice without affecting food intake and glucose homeostasis, and also reduced the plasma levels of total ghrelin and the expression of ghrelin O-acyltransferase in the stomach; however, the plasma levels of ghrelin and the ghrelin/total ghrelin ratio were unaffected. Furthermore, PF significantly increased the expression of growth hormone secretagogue receptor 1 alpha (GHSR1α, encoded by the *Ghsr* gene) in the intestine, whereas the levels of GHSR1α in the brain were only marginally downregulated following subchronic PF treatment. Finally, the genetic deletion of *Ghsr* attenuated the antidepressant-like effects of PF in mice exposed to CMS. These results suggested that increased GHSR1α expression in the intestine mediates the antidepressant-like effects of PF. Understanding peripheral ghrelin/GHSR signaling may provide new insights for the screening of antidepressant drugs that produce fast-acting and sustained effects.

## Introduction

Major depressive disorder (MDD) is a common mental illness that affects at least 322 million people and is expected to be the leading cause of burden of disease globally by 2030 (WHO, 2017; Saman et al., 2018). Significant progress has been made in the development of therapeutic agents, such as serotonin selective reuptake inhibitors (SSRIs), that demonstrate satisfactory efficacy and tolerability. However, fewer than half of all patients respond to currently available antidepressants. Moreover, SSRIs have a delayed onset of action and significant side-effects, including bodyweight gain, sexual dysfunction, and even an increased risk of suicide (Moret et al., 2009). The discovery that a single dose of ketamine can produce rapid antidepressant effects in people with MDD, including those with treatment-resistant symptoms, as well as recent investigations into ketamine efficacy, have provided novel neurological insights into the mechanisms underlying its antidepressant efficacy (Krystal et al., 2019). However, ketamine also has serious side-effects that limit its clinical use, including increased dissociative and other psychotomimetic symptoms, cardiovascular manifestations, cognitive impairment, and potential for abuse (Short et al., 2018). These issues emphasize the pressing need for the development of new classes of antidepressant medications with rapid onset of action, greater efficacies and tolerability, and longer-lasting effects (Yan et al., 2011).

The growth hormone secretagogue receptor 1 alpha (GHSR1α, encoded by *GHSR*) is a unique member of the G protein-coupled receptor family that comprises the largest class of pharmacological targets for current drugs and drug discovery (Campbell and Smrcka, 2018). GHSR1α is activated by ghrelin, which is generated following the octanoylation of the ghrelin (encoded by *GHRL*) precursor by ghrelin O-acyltransferase (GOAT, encoded by *MBOAT4*). Ghrelin binds to GHSR1α at multiple central and peripheral sites to modulate energy homeostasis, reproduction, cognition, reward, and emotion (Abizaid and Hougland, 2020). Recent studies have linked the ghrelin/GHSR system to the pathophysiology of MDD. A genome-wide DNA methylation profile analysis conducted on 34 monozygotic twins with depressive symptoms showed that *GHSR* DNA methylation was highly associated with depression (Cordova-Palomera et al., 2018). Although the clinical data for the link between ghrelin and MDD are not consistent, most studies have reported that the plasma level of ghrelin is significantly increased in patients with MDD, and is associated with a depressive status (Wittekind and Kluge, 2015; Algul and Ozcelik, 2018; Agawa et al., 2019). Animal studies have consistently found that the plasma levels of ghrelin are elevated in adult mice following chronic stress, including chronic social defeat stress (CSDS) and chronic mild stress (CMS) (Lutter et al., 2008; Chuang et al., 2011; Harmatz et al., 2017; Huang et al., 2017; Han et al., 2019). Moreover, peripheral ghrelin administration was shown to induce an antidepressant-like effect in mice during a forced swimming test (FST) (Lutter et al., 2008). In addition, exposure to CSDS elicited more severe depressive-like phenotypes in *Ghsr*-null (*Ghsr*^–/^*^–^*) mice or hippocampal-specific GHSR1α knockdown mice than in their respective control littermates (Lutter et al., 2008; Han et al., 2019). However, some studies have shown that chronic subcutaneous administration of ghrelin and growth hormone-releasing peptide 2, a synthesized agonist of GHSR1α, could not reverse the depressive-like behaviors induced by CSDS (Gupta et al., 2019). Meanwhile, in male rats, the central administration of ghrelin induced depressive-like behaviors during the FST and tail suspension test (Hansson et al., 2011; Jackson et al., 2019). Consequently, how ghrelin/GHSR signaling contributes to stress responses and mood regulation remains inconclusive.

Chinese herbal medicines have great potential as sources of new classes of antidepressants. Paeoniflorin (PF), a monoterpenoid glycoside compound, is the main active ingredient extracted from the root of *Paeonia lactiflora* Pall, also known as peony or Baishao, and is widely used in Chinese herbal formulae for the treatment of depressive-like disorders (Mao et al., 2008). Recent studies have shown that PF produces antidepressant-like effects in several animal models of depression, such as the CMS paradigm (Yu et al., 2019), as well as rat models of post-stroke depression (Hu et al., 2019) and menopausal depression (Huang et al., 2015). In this study, we sought to extend these findings by investigating the effects of PF in multiple rodent models of depression, focusing on its possible rapid-onset and long-lasting antidepressant-like effects. Additionally, we also explored how the ablation of GHSR1α would affect the anti-depressive response to PF. Our findings showed that PF produces rapid and sustained antidepressant-like effects. We found that PF treatment significantly increased the expression of GHSR1α in the intestine, but only marginally affected the levels of GHSR1α in the brain. In contrast, the genetic deletion of *Ghsr* attenuated the antidepressant-like effect of PF. These results suggested that the increased expression of GHSR1α in the intestine may mediate the antidepressant-like effects of PF. A comprehensive understanding of the properties of the GHSR system will facilitate the discovery of new classes of antidepressants, especially from traditional Chinese herbal medicines.

## Materials and Methods

### Animals

C57BL/6 male mice were obtained at 2–3 months of age from the Southern Medical University Animal Center (Guangzhou, China). The mice were housed in standard plastic rodent cages under standard laboratory conditions (24°C, 12-h light/dark cycle from 07:00 to 19:00) and with free access to food and water. Before the behavioral experiments, the mice were handled twice a day for at least 3 days. All the experiments were conducted following the Chinese Council on Animal Care guidelines. All the behavioral tests were conducted following the procedures established in our laboratory, with some modifications (Cao et al., 2013; Qin et al., 2019; Wang et al., 2019; Xiong et al., 2019).

The *Ghsr*^–/^*^–^* mice (C57BL/6 background) were provided by Dr. Jeffrey M. Zigman (University of Texas Southwestern Medical Center, Dallas, TX, United States). *Ghsr*^–/^*^–^* mice and their wild-type littermates were obtained from the incrossing of heterozygous *Ghsr*^+/^*^–^* mice, which were themselves derived from the crossing of *Ghsr*^–/^*^–^* and C57BL/6 mice. Genotyping was conducted as previously reported (Zigman et al., 2005).

### Drugs

PF (catalog# P0038), fluoxetine (catalog# F132), imipramine (catalog# PHR1797), and haloperidol (catalog# H1512) were purchased from Sigma–Aldrich Chemical Co. (St. Louis, MO, United States). Phenylmethylsulfonyl fluoride (PMSF) was purchased from Beyotime (Shanghai, China).

### Forced Swimming Test

For the FST, mice were intraperitoneally (i.p.) injected with PF (0.25, 0.5, 1, 2.5, 5, or 10 mg/kg), fluoxetine (20 mg/kg), imipramine (15 mg/kg), haloperidol (1 mg/kg), or saline. Thirty minutes after the injection, the mice were gently placed for 6 min in a commercial transparent glass cylinder (height 45 cm, diameter 19 cm) filled with water (22–25°C) to a height of 23 cm. The immobility time was recorded during the last 4 min by an observer who was blinded to the treatments. To test the sustained antidepressant-like effect of PF during the FST, a different group of mice was injected with PF (1 mg/kg) or saline daily for 7 consecutive days, and the behavioral tests were conducted 28 days later.

### Open Field Test

Mice were placed in an open chamber (40 cm × 40 cm × 30 cm) (Accuscan Instruments, Columbus, OH, United States) with transparent plastic walls and were allowed to freely explore the chamber for 10 min. The total distance traveled across the chamber during the last 5 min was analyzed using VersaMax software (Accuscan Instruments, Columbus, OH, United States).

### Novelty-Suppressed Feeding Test

The Novelty-Suppressed Feeding Test (NSF) was conducted following previous reports (Santarelli et al., 2003; Yan et al., 2010b). The mice were single-housed briefly and food-deprived for 24 h before the NSF test. Before the test, a normal chow diet pellet was placed in the center of a plastic black box (50 cm × 50 cm × 50 cm) over a 2-cm thick wooden bedding. The mice were introduced into a corner of the arena. The NSF test lasted within 5 min. The time the mice spent to take a bite of food (latency) was recorded by a trained observer. Immediately after one trial, the mice were returned to their home cage containing pre-weighed food pellets, and the amount of food consumed within 5 min was measured as the home cage food intake.

### Chronic Mild Stress Paradigm

The methodology for the CMS paradigm was as previously described (Cao et al., 2013; Qin et al., 2019; Xiong et al., 2019). Mice were briefly individually caged and habituated to water or a 1% sucrose solution for the assessment of the baseline sucrose preference. The mice underwent the sequential application of a variety of mild stressors, including restraint, a forced swim in ice-cold water, food and water deprivation, cage tilting (45°), and strobe lighting (Table 1). The schedule was repeated weekly. For the treatments, saline, PF (1 mg/kg), or imipramine (15 mg/kg) was injected daily for 4 weeks (Figure 3A). The control mice were kept under the same housing conditions but did not undergo stress treatment. Sucrose preference was measured weekly and the coat score was evaluated by an experienced observer.

**TABLE 1:** Chronic mild stress paradigm.

Day Stress	Monday	Tuesday	Wednesday	Thursday	Friday	Saturday	Sunday
Restraint	09:00–12:00		09:00–12:00				
Water deprivation	12:00–19:00			20:00 start	08:00 end		
Empty bottle	19:00 start	08:00 end					
Stroboscopic illumination		08:00–15:00			19:00 start	12:00 end	
Swim stress		15:00–19:00					
Soiled cage		20:00 start	09:00 end				
Food and water deprivation			14:00 start	09:00 end	08:00–13:00		
Cage tilting				09:00–15:00			09:00–19:00
Food deprivation						12:00 start	09:00 end
Sucrose test and coat score					13:00–14:00		
No stress	09:00 end			15:00–20:00			19:00 start

### Sucrose Preference Test

Mice were habituated for 4 days to either water or a 1% sucrose solution, as follows: On days 1 and 2, the mice were provided with two bottles, both of which were filled with water (w/w), while on days 3 and 4, both bottles were filled with a 1% sucrose solution (s/s) instead. The SPT was performed after the deprivation of food and water later (Table 1). For the SPT, one of the bottles (A) contained a 1% sucrose solution, and the other (B) contained water (s/w). The fluid that was consumed from each bottle within 1 h was measured weekly, and the sucrose preference was calculated as 100 × [Vol_A_ / (Vol_A_ + Vol_B_)].

### Coat Score Assay

The state of the fur was evaluated weekly, and the coat score was measured as the sum of the score of the following seven different body parts: head, neck, dorsal coat, ventral coat, tail, forepaws, and hindpaws. For each of the seven areas, a score of 1 was given for a well-groomed coat and the maximum score was 7.

### Blood Glucose Measurement

Mice were intraperitoneally injected with PF (1 mg/kg) or saline once a day for 7 consecutive days. The bodyweight and the food intake were measured daily before injection. The glucose tolerance test was conducted as previously reported (Barnett et al., 2010). Briefly, 6 h after the last PF injection (on day 7; Figure 4A), the mice were deprived of food for 18 h and then intraperitoneally injected with a single 2.5 g/kg dose of glucose (on day 8). Blood was sampled from the tail vein at 0, 15, 30, 60, and 120 min after glucose injection. Glucose was measured using blood glucose test strips and a glucose reading meter (BAYER Contour TS, Leverkusen, Germany).

### Quantitative Real-Time PCR (qPCR)

The tissues were quickly harvested from the mice injected with saline or PF (1 mg/kg) for 7 consecutive days. The total RNA was prepared using a homogenizer (Precellys Evolution, Bertin Technologies, France) and extracted using Trizol. cDNA was synthesized using the PrimeScript^TM^ RT Reagent Kit (Takara Biomedical Technology Co., Beijing, China). qPCR analysis was performed using the TB Green qPCR Master Mix (Takara) following the standard protocol and a 7500 Real-Time PCR System (Thermo Fisher Scientific, Waltham, MA, United States). *18S* was used as an internal control for normalization during the ΔΔC_t_ method. All the primers used are as follows: *Ghrl*, 5′–GACAGAGGAGGAGCTGGAGA–3′ and 5′–GGCCATGCTGCTGATACTGA–3′; *Mboat4*, 5-ATT TGTGAAGGGAAGGTGGAG-3′ and 5′-CAGGAGAGCAGGG AAAAAGAG-3; *Ghsr*, 5′– CTATCCAGCATGGCCTTCTC–3′ and 5′–AAGACGCTCGACACCCATAC–3′; *18S*, 5′– AGTTC CAGCACATTTTGCGAG–3′ and 5′– TCATCCTCCGTGAGTT CTCCA –3′.

### Western Blot Analysis

Duodenum was harvested from the mice that were injected daily with saline or PF (1 mg/kg) for 7 consecutive days and homogenated with RIPA buffer (Thermo Fisher Scientific) containing 1 mM PMSF. After centrifugation at 16,000 *g* for 20 min, the supernatant was collected and quantified using the BCA Protein Assay Kit (Thermo Fisher Scientific). Seventy-five micrograms of proteins were separated using 10% SDS-PAGE and transferred to PVDF membranes (PerkinElmer). The membranes were blocked using 5 % (w/v) skim milk for 1 h at room temperature, followed by incubation overnight at 4°C with the primary antibody anti-MBOAT4 (1:1,000; Bioss; catalog# bs-13355R) or anti-GHS-R1 (H–80) (1:1,000; catalog# sc-20748; Santa Cruz Biotechnology), and then incubated for 1h at room temperature with the secondary antibody peroxidase-conjugated AffiniPure goat anti-rabbit IgG (H+L), or and goat anti-mouse IgG (H+L) (1:5000, ZSGB-Bio, Beijing, China). The protein levels were normalized to glyceraldehyde-3-phosphate dehydrogenase (anti-GHPDH, 1:5,000; catalog# 60004-1-Ig; Proteintech Group, Rosemont, IL, United States) on the same gel.

### Automated Capillary Electrophoresis Western Blot Analysis

The experiments were conducted following the user guide of ProteinSimple. The brain tissues were homogenized, quantified, and mixed at a 4:1 ratio with 5 × Master. The samples were incubated at 95°C for 5 min for protein denaturation and reduction. The treated samples and the ladder (containing six proteins with molecular weights of 12, 40, 66, 116,180, and 230 kDa) (catalog# PS-ST01-8, ProteinSimple Biosciences & Technology Co., San Jose, CA, United States) were loaded into individual wells of the same row in a 384-well plate. The primary antibody, blocking solution (antibody diluent), stacking matrix, separation matrix (catalog# DM–001, ProteinSimple) and secondary antibody were loaded into the same 384-well plate. The automated western blot was performed using the WESTM instrument (ProteinSimple) with single-use silica capillaries (5 cm by length and 100 μm by inner diameter). The proteins were separated by electrophoresis at 375 V for 25 min into individual sets of 25 capillaries. The capillaries were incubated with antibody diluent for 5 min to block the non-specific binding of the antibody. After the primary antibody was incubated for 30 min, the secondary antibody was introduced into the capillary lumen for 30 min. The protein levels were normalized to the levels of β-actin (1:500; catalog# 20536–1–AP; Proteintech) on the same plate.

### Detection of Plasma Ghrelin by ELISA

The mice were injected daily with saline or PF (1 mg/kg) for 7 consecutive days. The blood samples were obtained from the orbit and transferred immediately into tubes with 50 mM EDTA, which was followed by centrifuging at 3,000 *g* for 10 min at 4°C. Fifty microliters of plasma were separated into tubes containing 1 μl 100 mM PMSF and 2.5 μl 1 N HCl, and they were stored at –80°C. The collections were staggered to match the controls with the treated samples. The plasma levels of total ghrelin (including ghrelin and desacyl-ghrelin) and ghrelin were measured using the enzyme immunoassay kits (catalog# EZRGRT–91K, catalog# EZRGRA–90K, Millipore, Hayward, CA, United States).

### Statistical Analysis

The data were expressed as means ± SEM (standard error of the mean) using Graphpad Prism 8.4.2 (GraphPad Software, La Jolla, CA, United States). When equal variances were assumed following a test for homogeneity of variance, the two-tailed Student’s *t*-test (for two-group comparisons) or one-way ANOVA (for multiple-group comparisons) was used to evaluate whether differences were significant. Dunnett’s T3 test for *post hoc* comparisons was used when equal variances were not assumed. Two-way ANOVA with Bonferroni’s multiple comparisons test was used for repeated measures. *P* < 0.05 denoted statistical significance.

## Results

### PF Elicited Long-Lasting Antidepressant-Like Effects in the FST

**FIGURE 1:**
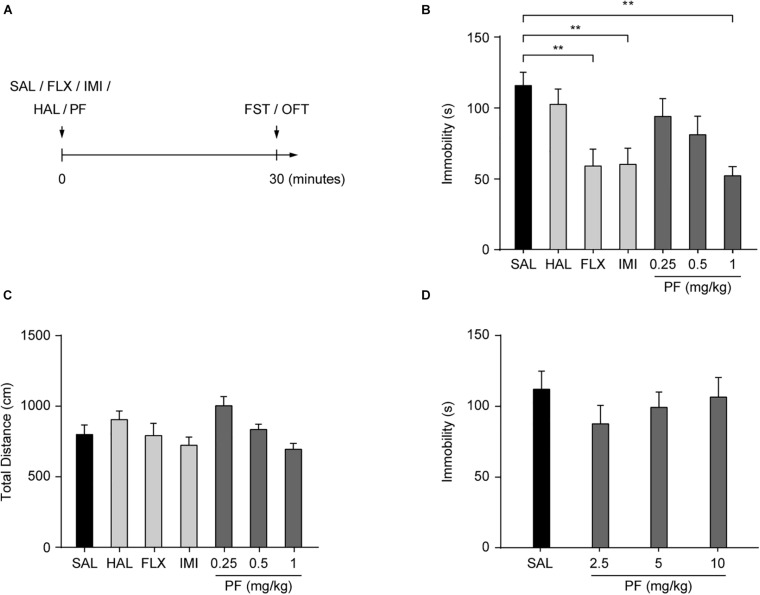
Paeoniflorin (PF) produced antidepressant-like effects in the forced swimming test (FST). **(A)** Schematic diagram of the experimental design. **(B)** Duration of immobility in the FST (*n* = 8–10). **(C)** Total distance traveled during the open field test (OFT) (*n* = 8–10). **(D)** Duration of immobility in the FST after treatment with PF at the dosage of 2.5, 5, or 10 mg/kg (*n* = 10). SAL, saline; FLX, fluoxetine; IMI, imipramine; HAL, haloperidol. The data are expressed as means ± SEM. **P < 0.01.

Adult C57BL/6 mice were injected (i.p.) with PF or saline. After 30 min, the FST, a well-established method for screening novel candidate antidepressants (Yan et al., 2010a), was performed to assess the antidepressant effects of PF (Figure 1A). Two classical antidepressants, fluoxetine and imipramine, were used as positive controls, while haloperidol, an antipsychotic drug, was used as the negative control. Consistent with our previous reports (Yan et al., 2010b; Zhu et al., 2010), fluoxetine and imipramine decreased the duration of immobility during the FST, whereas haloperidol had no effect. Meanwhile, PF treatment (0.25, 0.5, or 1 mg/kg) significantly and dose-dependently decreased the duration of immobility (*n* = 8–10, *F*_[6,54]_ = 4.3620, *P* = 0.0012; one-way ANOVA), but did not affect locomotor activity (*n* = 8–10, *F*_[6,54]_ = 3.1122, *P* = 0.0108, one-way ANOVA) (Figures 1B,C). This indicated that PF can produce an antidepressant-like effect. However, at higher PF concentrations (2.5–10 mg/kg), no antidepressant-like effect was observed (*n* = 10, *F*_[3,36]_ = 0.6860, *P* = 0.5665, one-way ANOVA) (Figure 1D). Overall, these results were generally consistent with those recently reported (Yu et al., 2019). Subsequently, PF was used at a dosage of 1 mg/kg in this study.

**FIGURE 2:**
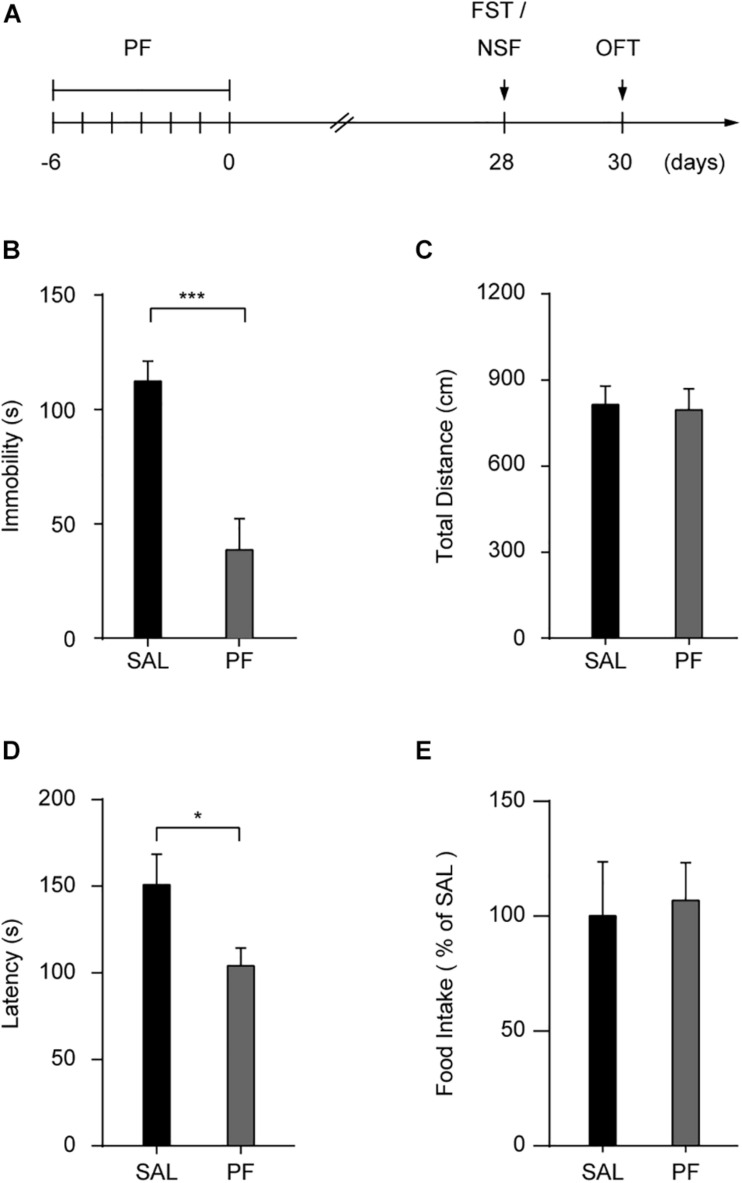
Paeoniflorin (PF) produced sustained antidepressant-like effects in the forced swimming test (FST). **(A)** Schedule for detecting the long-lasting antidepressant-like effect of PF. **(B,C)** Statistical data for the FST **(B)** and the open field test (OFT) **(C)** conducted 28 days after subchronic PF treatment (*n* = 10). **(D,E)** The latency **(D)** and the food intake **(E)** in the novelty suppressed feeding test (*n* = 7–8). SAL, saline. The data are expressed as means ± SEM. *P < 0.05, ***P < 0.001.

To determine whether PF treatment could produce a sustained antidepressant-like effect, mice were treated with saline or PF for 7 consecutive days and their behaviors were analyzed after 28 days (Figure 2A). This subchronic PF treatment significantly decreased the duration of immobility in the FST after 28 days, whereas locomotion was not affected in the open field test (*n* = 10, *t*_FST [18]_ = 4.5430, *P* = 0.0003; *t*_OFT [18]_ = 0.1887, *P* = 0.8525; two-tailed unpaired *t*-test) (Figures 2B,C). Moreover, in a different group of mice, the 7–day treatment with PF shortened the latency to feed in the novelty-suppressed feeding test (NSF) relative to that with saline treatment (*n* = 7–8, *t*_[13]_ = 2.2077, *P* = 0.0458; two-tailed unpaired *t*-test) (Figure 2D). There was no difference in food consumption between these two groups when the mice were returned to their home cages (*n* = 7–8, *t*_[13]_ = -0.2258, *P* = 0.8249; two-tailed unpaired *t*-test) (Figure 2E), indicating that the reduced latency to feed observed following PF administration was not likely to have been due to a change in appetite.

### PF Rapidly Reversed the Depressive Phenotypes Induced by CMS

**FIGURE 3:**
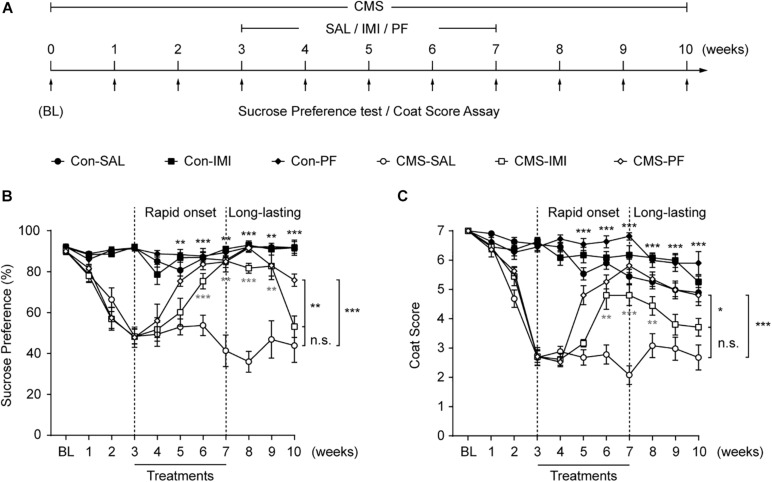
The rapid and sustained antidepressant-like effect of paeoniflorin (PF) during chronic mild stress (CMS). **(A)** Schematic diagram of the experimental design for the treatments with PF, imipramine (IMI), and saline (SAL) during exposure to CMS. **(B,C)** The results of the sucrose preference test **(B)** and the coat score assay **(C)** during exposure to CMS (*n* = 10–11). The data are expressed as means ± SEM. *P < 0.05, **P < 0.01, ***P < 0.001; n.s., not significant.

We next determined whether PF could reverse the depressive-like behaviors induced by chronic stress. For this, mice were subjected or not (control) to CMS for 10 weeks, and PF was administered for four weeks starting at the end of the third week (Figure 3A). Imipramine was used as a positive control. In control mice, imipramine and PF did not affect the sucrose preference or coat score index when compared with saline. Consistent with our previous observations (Cao et al., 2013; Qin et al., 2019), the mice subjected to CMS exhibited progressive anhedonia, as evidenced by a gradual reduction in sucrose preference and the deterioration in the coat condition. In contrast, four weeks of imipramine treatment reversed the CMS-induced anhedonia phenotype, an effect that could already be observed after three weeks. Meanwhile, with PF, an improvement in the depressive-like behaviors of the mice was apparent after only two weeks of treatment (*n* = 10–11, *F _*sucrose preference*_*_[5,59]_ = 79.6300, *P* < 0.001; *F _*Coat score*_*_[5,59]_ = 117.5000, *P* < 0.001; repeated-measures ANOVA) (Figures 3B,C).

As MDD has a high rate of recurrence (Malhi and Mann, 2018), we sought to determine whether PF could exert a long-lasting antidepressant-like effect during exposure to CMS. For this, treatment was discontinued at the end of the seventh week, while the CMS procedures were continued (Figure 3A). Under CMS, the antidepressant-like effect of imipramine lasted only for two weeks, as evidenced by the marked decrease in sucrose preference and coat score index of the mice in the CMS-imipramine group. Meanwhile, three weeks after drug withdraw, no significant changes in sucrose preference or coat score were observed in the CMS-imipramine group when compared with CMS-saline group. In contrast, the antidepressant-like effect of PF lasted for at least three weeks after the discontinuation of PF treatment (Figures 3B,C). Together, these results indicated that PF produced rapid and sustained antidepressant-like effects in mice subjected to CMS.

### PF Treatment Decreased Bodyweight Gain

**FIGURE 4:**
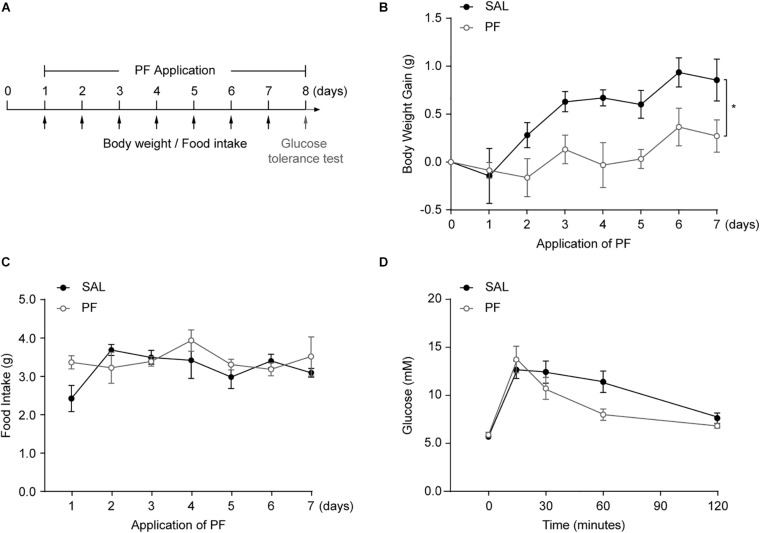
The effects of paeoniflorin (PF) on metabolic status. **(A)** Schematic representation of the experimental design (*n* = 8). **(B)** Bodyweight gain following PF/saline (SAL) administration. **(C)** Cumulative food intake. **(D)** Plasma glucose levels during the glucose tolerance test. The data are expressed as means ± SEM. *P < 0.05.

MDD is associated with alterations in appetite and bodyweight, and mounting evidence indicates that obesity increases the risk of MDD, and *vice versa* (Chuang et al., 2011; Mills et al., 2019). To detect whether PF could influence metabolic status, mice were injected daily with PF for 7 consecutive days, and bodyweight gain and food intake were evaluated (Figure 4A). As shown in Figure 4B, PF administration significantly decreased bodyweight gain compared with saline treatment (*n* = 8, *F*_[1,14]_ = 6.2130, *P* = 0.0258; repeated-measures ANOVA); however, there was no difference in food intake between the two groups (*n* = 8, *F*_[1,14]_ = 1.1080, *P* = 0.3519; repeated-measures ANOVA) (Figure 4C), indicating that PF did not affect appetite. To determine whether PF affected glucose homeostasis, mice received a glucose challenge (2.5 g/kg, i.p.) after subchronic PF administration (Figure 4A). As previously reported (Barnett et al., 2010), this glucose challenge significantly increased plasma glucose levels. In contrast, PF had little effect on the increased glucose responses when compared with saline group (*n* = 8, *F*_[1,14]_ = 1.1830, *P* = 0.2951; repeated-measures ANOVA) (Figure 4D).

### PF Affected the Peripheral Ghrelin/GHSR System

**FIGURE 5:**
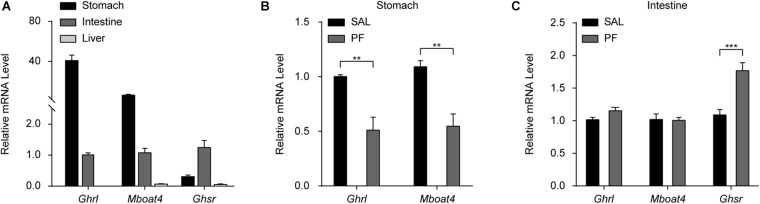
The effects of paeoniflorin (PF) on the mRNA levels of factors in the peripheral ghrelin/GHSR system. **(A)** qPCR analysis of *Ghrl*, *Mboat4*, and *Ghsr* mRNA in the stomach, intestine, and liver in adult C57BL/6 mice (*n* = 8). **(B)** The effects of the 7-day treatment with PF or saline (SAL) on the mRNA levels of *Ghrl* and *Mboat4* in the stomach (*n* = 8). **(C)** The effects of PF or SAL on the mRNA levels of *Ghrl*, *Mboat4*, and *Ghsr* in the intestine (*n* = 8). The data are expressed as means ± SEM. **P < 0.01, ***P < 0.001.

Ghrelin/GHSR signaling is one of the two well-characterized systems linking energy homeostasis with mood regulation (Chuang et al., 2011; Cui et al., 2017). The components of the ghrelin/GHSR system—ghrelin, GOAT, and the liver-expressed antimicrobial peptide 2 (LEAP2)—are mainly expressed in peripheral organs, whereas GHSR1α is primarily found in the central nervous system (CNS) (Zigman et al., 2006; Abizaid and Hougland, 2020). Increasing evidence suggests that signaling changes in peripheral organs in response to chronic stress, such as those observed in the liver–brain axis and gut–brain axis, may contribute to the pathophysiology of MDD (Zheng et al., 2016; Qin et al., 2019). Therefore, to investigate the mechanism underlying the antidepressant-like effect of PF, we focused on examining its effect on the ghrelin/GHSR system in peripheral organs, especially the gastrointestinal tract. Using qPCR analysis, we found that ghrelin and GOAT mRNA was highly expressed in the stomach of adult C57BL/6 mice, but was undetectable in the liver of the same animals. Meanwhile, *Ghsr* mRNA was predominately expressed in the intestine (Figure 5A). These results were generally consistent with those previously reported (Gahete et al., 2013). To further examine the effect of PF, mice were treated with PF for 7 consecutive days, following which the tissues were collected for qPCR, ELISA, and western blot analysis. PF treatment for 7 days significantly decreased the mRNA levels of *Ghrl* and *Mboat* in the stomach (*n* = 8, *t _*Ghrl*_*_[14]_ = 4.0638, *P* = 0.0044; *t _*Mboat4*_*_[14]_ = 4.3756, *P* = 0.0013; two-tailed unpaired *t*-test) (Figure 5B). In contrast, although PF treatment had little effect on the intestinal mRNA levels of *Ghrl* and *Mboat*, that of *Ghsr* increased after treatment with PF when compared with saline treatment group (*n* = 8, *t _*Ghrl*_*_[14]_ = -2.1235, *P* = 0.0520; *t _*Mboat4*_*_[14]_ = 0.1473, *P* = 0.8850; *t _*Ghsr*_*_[14]_ = -4.5746, *P* = 0.0004; two-tailed unpaired *t*-test) (Figure 5C).

**FIGURE 6:**
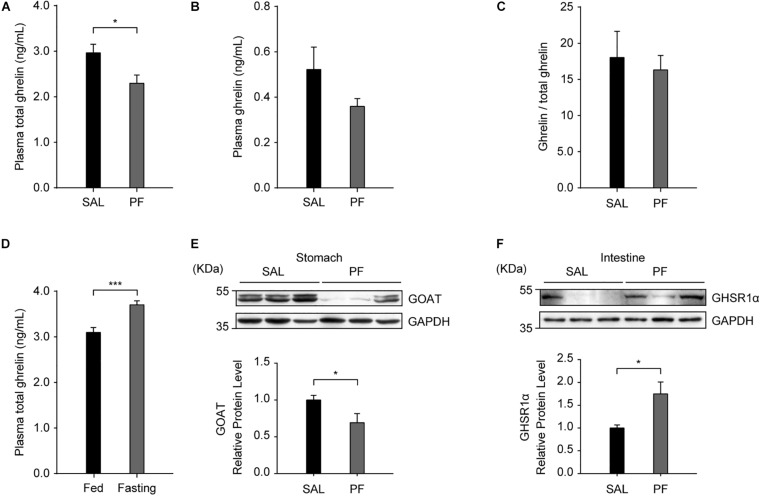
The effects of paeoniflorin (PF) on the levels of proteins in the peripheral ghrelin/GHSR system. **(A)** Compared with saline (SAL) treatment, 7-day treatment with PF decreased the plasma levels of total ghrelin (*n* = 8). **(B)** The plasma levels of ghrelin in C57BL/6 mice treated with PF or SAL for 7 consecutive days (*n* = 8). **(C)** The ghrelin/total ghrelin ratio in the plasma following subchronic treatment with PF or SAL (*n* = 8). **(D)** The plasma levels of total ghrelin in adult C57BL/6 mice after 24 h of fasting (*n* = 10). **(E)** The protein levels of GOAT in the stomach after 7 days of treatment with PF or SAL (*n* = 8). **(F)** The protein levels of GHSR1α in the intestine were higher with PF administration than with SAL administration (*n* = 8). The data are expressed as means ± SEM. *P < 0.05, ***P < 0.001.

Ghrelin is produced by neuroendocrine cells (named “X/A-like” in mice and “P/D1” in humans) in the oxyntic mucosa of the gastric fundus (Muller et al., 2015; Zhang et al., 2020), and the main route for conveying the gastric-derived ghrelin signal to the brain is the blood circulation (Yanagi et al., 2018). Consequently, to determine the role of ghrelin in the antidepressant effect of PF, we examined the effect of PF on the plasma level of ghrelin. We found that, compared with saline treatment, PF administration significantly decreased the plasma level of total ghrelin (*n* = 8, *t*_[14]_ = 2.5384, *P* = 0.0236; two-tailed unpaired *t*-test) (Figure 6A), but had no effect on the plasma levels of ghrelin (*n* = 8, *t*_[14]_ = 1.2625, *P* = 0.2274; two-tailed unpaired *t*-test) or the ghrelin/total ghrelin ratio (*n* = 8, *t*_[14]_ = 1.2218, *P* = 0.2420; two-tailed unpaired *t*-test) (Figures 6B,C). Consistent with a previous report (Lutter et al., 2008), the plasma levels of total ghrelin increased when the mice were fasted for 24 h (*n* = 10, *t*_[18]_ = -4.3686, *P* = 0.0004; two-tailed unpaired *t*-test) (Figure 6D), thereby validating the ELISA used for detecting blood ghrelin levels. Moreover, consistent with the qPCR results, western blot analysis showed that GOAT protein levels in the stomach were decreased after PF treatment when compared with the controls (*n* = 8, *t*_[14]_ = 2.2165, *P* = 0.0437; two-tailed unpaired *t*-test) (Figure 6E). In contrast, the protein levels of GHSR1α in the intestine were increased 1.75-fold following PF administration (*n* = 8, *t*_[14]_ = -2.7502, *P* = 0.0253; two-tailed unpaired *t*-test) (Figure 6F). Taken together, these results suggested that GHSR signaling in the intestine may be involved in the antidepressant-like effects of PF.

### PF Treatment Had Little Effect on the Levels of GHSR1α in the Brain

**FIGURE 7:**
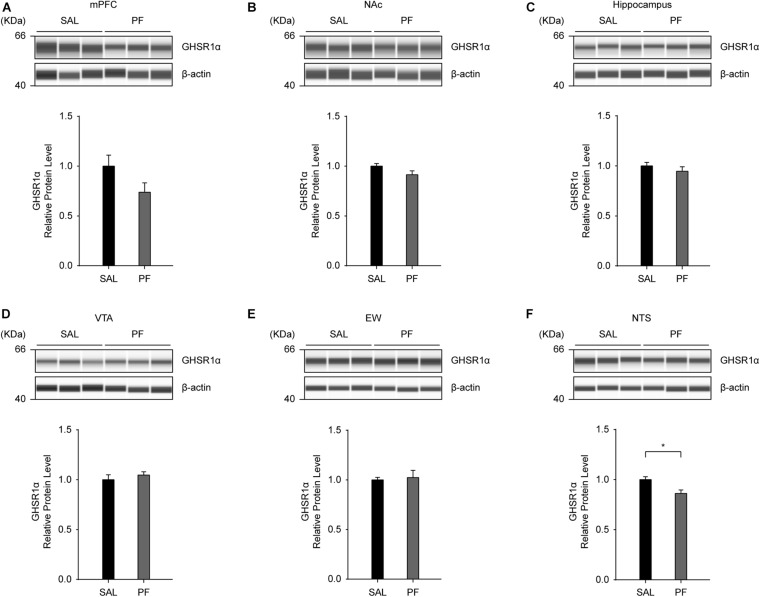
The effects of paeoniflorin (PF) on the levels of GHSR1α in the brain. **(A–E)** Seven days of PF treatment did not affect the protein levels of GHSR1α in the medial prefrontal cortex (mPFC) **(A)**, nucleus accumbens (NAc) **(B)**, hippocampus **(C)**, ventral tegmental area (VTA) **(D)**, or Edinger–Westphal nucleus (EW) **(E)** (*n* = 8) when compared with saline (SAL) treatment. **(F)** Subchronic PF treatment decreased the protein levels of GHSR1α in the nucleus tractus solitarii (NTS) (*n* = 8). The data are expressed as means ± SEM. *P < 0.05.

To examine the effect of PF on the levels of GHSR1α in the brain, we employed an automated capillary electrophoresis-based western blotting strategy. We selected four brain areas that have been implicated in the pathogenesis of MDD, including the medial prefrontal cortex (mPFC), the nucleus accumbens (NAc), the hippocampus, and the ventral tegmental area (VTA) (Russo et al., 2012; Malhi and Mann, 2018); as well as two other sites that display high GHSR1α expression according to the Allen Brain Atlas^1^, namely, the Edinger–Westphal nucleus (EW) and the nucleus tractus solitarii (NTS). As shown in Figure 7, PF only marginally altered the expression of GHSR1α in these brain areas, except for the NTS, where a significant reduction in the levels of GHSR1α was observed following PF administration (*n* = 8, *t _*mPFC*_*_[14]_ = 1.8162, *P* = 0.0994; *t _*NAc*_*_[14]_ = 1.8485, *P* = 0.0943; *t _*hippocampus*_*_[14]_ = 0.9330, *P* = 0.3728; *t _*VTA*_*_[14]_ = -0.7773, *P* = 0.4550; *t _*EW*_*_[14]_ = -0.2906, *P* = 0.7773; *t _*NTS*_*_[14]_ = 0.4330, *P* = 0.0119; two-tailed unpaired *t*-tests).

### The Deletion of *Ghsr* Attenuated the Antidepressant-Like Effect of PF

**FIGURE 8:**
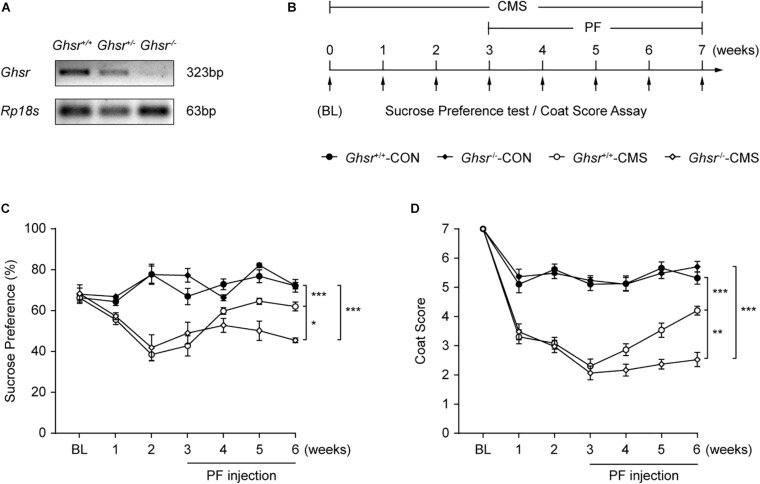
The deletion of *Ghsr* attenuated the antidepressant-like effect of paeoniflorin (PF). **(A)** Genotyping shows the downregulation of *Ghsr* mRNA in *Ghsr*^–/^*^–^* mice and their heterozygous littermates (*Ghsr*^+/^*^–^*) compared with the wild-type controls (*Ghsr*^+/+^). **(B)** Schematic diagram of the experimental design. **(C,D)** The sucrose preference test and coat score assay following exposure to chronic mild stress (CMS) (*n* = 6, 7, 9, and 10 for *Ghsr*^+/+^-CON (control; not exposed to CMS), *Ghsr*^–/^*^–^*-CON, *Ghsr*^+/+^-CMS, and *Ghsr*^–/^*^–^*-CMS, respectively). The data are expressed as means ± SEM. *P < 0.05, **P < 0.01, ***P < 0.001; n.s., not significant.

Finally, we determined whether the deletion of *Ghsr* could attenuate the antidepressant-like effect of PF. After genotyping (Figure 8A), *Ghsr*^–/^*^–^* mice and their wild-type (*Ghsr*^+/+^) littermates were subjected or not (controls) to CMS and then administered PF or saline after the third week (Figure 8B). After three weeks of CMS, both *Ghsr*^–/^*^–^* mice and their wild-type littermates exhibited anhedonia. However, PF administration could reverse the depressive-like behaviors of the *Ghsr*^+/+^ mice exposed to CMS, as evidenced by the improved sucrose preference and coat condition. In contrast, the antidepressant-like effect of PF was attenuated in *Ghsr*^–/^*^–^* mice. Meanwhile, PF had little effect on the behaviors of mice in the control groups (*n* = 6–10, *F _*Sucrose preference*_*_[3,27]_ = 60.9048, *P* < 0.001; *F _*Coat score*_*_[3,27]_ = 164.8719, *P* < 0.001; repeated-measures ANOVA) (Figures 8C,D).

## Discussion

This study was designed to identify targets for use in screening new classes of antidepressant drugs using PF as a chemical probe. The major findings were as follows: First, the administration of PF produced a rapid and sustained antidepressant-like effect during the FST and following exposure to CMS in adult mice. Second, PF significantly increased the expression level of GHSR1α in the intestine, while only marginally decreasing its expression in the brain. In contrast, PF treatment decreased the plasma levels of total ghrelin without affecting the plasma levels of ghrelin. Third, the genetic deletion of *Ghsr* attenuated the antidepressant-like effect of PF. Our results, combined with those of previous reports showing that the peripheral administration of ghrelin induces an antidepressant-like effect while the central infusion of ghrelin results in depressive-like behaviors (Lutter et al., 2008; Hansson et al., 2011; Jackson et al., 2019), suggest that a comprehensive understanding of the mechanisms underlying peripheral ghrelin/GHSR signaling may provide new insights for screening antidepressants that elicit fast-acting and sustained effects.

Ghrelin/GHSR signaling is one of the two well-characterized systems (the other being leptin signaling) linking energy homeostasis to mood regulation (Cui et al., 2017). In the initial experiment, we found that PF treatment not only produced an antidepressant-like effect, but also decreased the bodyweight gain of mice without affecting food intake or glucose homeostasis. Additionally, the components of the ghrelin/GHSR system, which include ghrelin, GOAT, and LEAP2, are mainly expressed in peripheral organs, especially the gastrointestinal tract (Abizaid and Hougland, 2020). For instance, ghrelin is mainly a stomach-derived hormone, GOAT is mainly expressed in the stomach, and LEAP2, an endogenous GHSR antagonist, is expressed predominantly in the liver (Gahete et al., 2013; Mani et al., 2019). Moreover, increasing evidence suggests that signaling changes in peripheral organs in response to chronic stress, such as those observed in the liver–brain axis and gut–brain axis, may contribute to the pathophysiology of MDD (Zheng et al., 2016; Qin et al., 2019). Studies investigating the gut–brain axis have also demonstrated that intestinal microbes play a critical role in the modulation of behavior, particularly mood. For instance, a study surveying the microbiome population in a large cohort showed that two bacterial genera—*Coprococcus* and *Dialister*—were depleted in the microbiomes of patients with MDD, while the genus *Butyricicoccus* was linked to antidepressant treatment (Valles-Colomer et al., 2019). Moreover, a preclinical study showed that intestinal microbial remodeling using fecal microbiota transplantation derived from patients with MDD could induce depressive-like behaviors in germ-free mice (Zheng et al., 2016). In addition, chronic treatment with prebiotic fructooligosaccharides and galactooligosaccharides elicited antidepressive and anxiolytic effects in adult mice (Burokas et al., 2017). These results suggest that a causal relationship exists between intestinal microbiota and depressive phenotypes (Fung et al., 2017). However, the key regulator in the gut–brain axis remains unknown. GHSR1α is ubiquitously expressed throughout the gut–brain axis, including in the NTS and thalamic paraventricular nucleus, and represents a major therapeutic target for several disorders, including obesity, diabetes, anxiety, and depression. Consistent with these observations, in the present study, we found that GHSR1α was highly expressed in the intestine. Interestingly, it has been shown that microbiota-derived metabolites, such as short-chain fatty acids and lactate, can modulate ghrelin-mediated GHSR1α internalization in HEK293 cells transfected with GHSR1α (Torres-Fuentes et al., 2019). Meanwhile, another study reported that PF treatment could modulate the composition of intestinal microbiota that enhanced its antidepressant-like effects (Yu et al., 2019). In this study, we found that PF significantly increased the expression of GHSR1α in the intestine, whereas the genetic deletion of *Ghsr* attenuated the antidepressant-like effect of PF. These results indicate that increased intestinal GHSR1α expression may mediate the antidepressant-like effect of PF. In addition, increasing evidence has shown that fecal microbes and metabolites are also associated with changes in ghrelin expression and activity (Schalla and Stengel, 2020). Further studies are needed to determine whether intestinal GHSR1α signaling plays an important role in the gut–brain axis.

Ghrelin is secreted from ghrelin-producing cells that are predominantly located in the gastrointestinal tract (Abizaid and Hougland, 2020). Preproghrelin, the ghrelin precursor, undergoes endoproteolytic processing and posttranslational modifications to produce ghrelin (Kojima and Kangawa, 2005; Zhu et al., 2006; Collden et al., 2017). Moreover, ghrelin has an unusual Ser3 octanoylation, meaning that only ghrelin, and not desacyl ghrelin, can bind and activate GHSR1α (Barnett et al., 2010; Yanagi et al., 2018). Studies have shown that ghrelin-producing cells express high levels of β1 adrenergic receptors (β1-ARs). Genetic deletion of β1ARs in ghrelin-producing cells or the application of atenolol, a β1-AR-specific antagonist, decreased the plasma levels of both ghrelin and total ghrelin (Mani et al., 2016; Gupta et al., 2019), suggesting that the sympathoadrenal system is involved in the regulation of ghrelin production. Meanwhile, it has also been reported that PF might act on peripheral β1-ARs (Ohta et al., 1993). In the present study, we found that PF administration significantly decreased the plasma levels of total ghrelin, indicating that PF may act on gastric ghrelin-producing cells, thereby leading to a reduction in plasma ghrelin levels, either directly or indirectly. Moreover, we found that *Ghsr* mRNA levels in the stomach were decreased following PF treatment, further supporting the possibility that PF may modulate ghrelin expression in the stomach. In addition, GOAT protein levels in the stomach were decreased by the administration of PF, although the plasma levels of ghrelin and the ghrelin/total ghrelin ratio were not affected. This suggested that PF may impair ghrelin octanoylation, resulting in increased ghrelin degradation, thereby also contributing to lower plasma total ghrelin levels after PF treatment (Fetissov et al., 2017). Changes in ghrelin expression or activity can lead to bodyweight changes (Schalla and Stengel, 2020). Consistent with this, we found that PF treatment decreased the bodyweight gain of mice. Because PF may act on the sympathoadrenal system, and because studies have shown that sympathoadrenal activity and ghrelin can both regulate blood glucose levels (Mani et al., 2016), we examined the effect of PF on glucose metabolism. Interestingly, we found that PF administration had little effect on plasma glucose levels. These characteristics of PF may increase its potential for use as a novel antidepressant.

GHSRs are expressed in both peripheral tissues and CNS. In the brain, GHSR1α is enriched in the EW, VTA, hippocampus, and hypothalamus (Zigman et al., 2006). Mice that express functional GHSR1α only in dopaminergic neurons (located in the VTA and some parts of the hypothalamus, including the PVN and arcuate nucleus) displayed food-reward behavior in response to prolonged psychosocial stress, as well as increased food intake after ghrelin stimulation (Chuang et al., 2011), suggesting that GHSR1α in dopaminergic neurons is involved in the modulation of food intake, especially in response to stress. However, in the present study, we found that PF had no effect on food intake. We also found that the expression of GHSR1α in the VTA was unaffected by PF treatment, indicating that the effect of PF on the CNS likely did not play a role in the antidepressant-like effect of PF.

In summary, we demonstrated that PF produced a rapid and sustained antidepressant-like effect in multiple animal models of depression, possibly through increasing GHSR1α expression in the intestine. Our data suggested that therapeutic targeting of the ghrelin/GHSR system in the periphery may serve as a new approach for screening antidepressant drugs that produce fast-acting and sustained effects. Further experiments are needed to address how PF regulates ghrelin/GHSR signaling.

## Data Availability Statement

The original contributions presented in the study are included in the article/supplementary material, further inquiries can be directed to the corresponding author.

## Ethics Statement

The animal study was reviewed and approved by Chinese Council on Animal Care guidelines. Written informed consent was obtained from the owners for the participation of their animals in this study.

## Author Contributions

X-HZ designed the research. X-HZ, M-ZZ, and YZ analyzed the data and wrote the manuscript. M-ZZ, YZ, and Y-NZ conducted the behavioral tests. M-ZZ and YZ collected the tissue and blood samples. M-ZZ and X-HQ performed the drug administration, ELISA experiments, and the glucose tolerance test. YZ performed the qRT-PCR and western blotting. X-HQ bred the *Ghsr*-null mice and performed genotyping. All authors contributed to the article and approved the submitted version.

## Conflict of Interest

The authors declare that the research was conducted in the absence of any commercial or financial relationships that could be construed as a potential conflict of interest.

## Footnotes

1 http://mouse.brain-map.org/experiment/show/69257319
